# Issues Related to the Effectiveness of Extracorporeal Shock Wave Therapy for the Management of Lateral Elbow Tendinopathy

**DOI:** 10.3390/jcm11185413

**Published:** 2022-09-15

**Authors:** Dimitrios Stasinopoulos

**Affiliations:** Department of Physiotherapy, Faculty of Health and Caring Sciences, University of West Attica, Agiou Spyridonos 28, Egaleo, 12243 Athens, Greece; dstasinopoulos@uniwa.gr

One of the most popular recommended physical therapy modalities for the management of lateral elbow tendinopathy (LET) is extracorporeal shock wave therapy (ESWT). Three recently published systematic reviews (SRs) and meta-analysis (MA) on the effectiveness of ESWT for the management of LET revealed contradictory results [1,2,3]. Two of them reported superior outcomes of the ESWT compared with placebo ESWT or other modalities in the relief of pain and functional impairment (grip strength) [1,2]. Meanwhile, Karanasios et al. (2021) [3] found that ESWT presents no clinical benefits compared with placebo ESWT or treatment therapy in elbow disability, grip strength and pain intensity at follow-up occasions. The above difference occurred due to the many methodological issues found in the previously reported SRs and MA.

However, it is not time to stop conducting SRs and MA with the aim of determining the effectiveness of ESWT in the management of LET. Many issues can be evaluated in future SRs and MA in order to determine whether ESWT is an effective treatment approach in the therapy of LET

Although many physical therapy techniques have been used in the management of LET, the most effective physiotherapy treatment for LET is an exercise program, in the clinic or supervised [4]. ESWT is used as a supplement to an exercise program and not as a substitute for exercise [5]. According to this, the future SR and MA will determine the effectiveness of ESWT combined with exercise programs in individuals with LET.

In addition, LET is an appropriate term for clinical diagnosis when it refers to a painful tendon overuse tendon [6]. On the other hand, LET is not an appropriate term for clinical diagnosis when there is persistent LET (PLET) [7]. Lateral elbow pain syndrome (LEPS) seems to be the most appropriate clinical diagnostic term for PLET, since this references a variety of lateral elbow pain diagnoses, such as nerve involvement, neck/thoracic dysfunction, myofascial trigger points and tendinopathy, all involved in PLET [7]. Therefore, the effectiveness of ESWT in patients with LEPS should be evaluated.

Furthermore, ESWT is a dose–response modality, and the optimal treatment dose (ESWT parameters such as focused or radial, energy density, impulses, frequency, number of sessions, application method (scanning the whole area or only on the pain point), specific disease condition (calcification or not, acute or chronic) and anesthesia or not) has obviously not yet been found in order to be used in rehabilitation protocols [5]. Furthermore, SR and MA are needed to determine the appropriate protocol.

Moreover, ESWT is usually used when patients’ symptoms exist for more than 6 months or when all other types of conservative therapy fail [5]. The duration of LET/LEPS symptoms associated with the efficacy of ESWT should be determined in a future SR and MA.

Finally, future SR and MA should assess whether the improvements in outcome measures (pain, function, etc.) remain in those patients with longer follow-up. Answering this question would eliminate the self-limiting natural history of the condition or the placebo effect related to participation in a trial [8].

According to the above issues, the effectiveness of ESWT for LET/LEPS deserves more attention, and more research is needed to guide clinical practice.

Overall, I believe that the issues discussed in this editorial might help clinicians to improve their clinical practice. It is the intention of the author to generate questions about the optimal ESWT approach for the therapy of LET/LEPS.

## Data Availability

Not applicable.
